# Multiscale dynamical cross-talk in zeolite-catalyzed methanol and dimethyl ether conversions

**DOI:** 10.1093/nsr/nwac151

**Published:** 2022-08-04

**Authors:** Shanfan Lin, Yuchun Zhi, Zhiqiang Liu, Jiamin Yuan, Wenjuan Liu, Wenna Zhang, Zhaochao Xu, Anmin Zheng, Yingxu Wei, Zhongmin Liu

**Affiliations:** National Engineering Laboratory for Methanol to Olefins, Dalian National Laboratory for Clean Energy, Collaborative Innovation Center of Chemistry for Energy Materials (iChEM), Dalian Institute of Chemical Physics, Chinese Academy of Sciences, Dalian 116023, China; Energy College, University of Chinese Academy of Sciences, Beijing 100049, China; National Engineering Laboratory for Methanol to Olefins, Dalian National Laboratory for Clean Energy, Collaborative Innovation Center of Chemistry for Energy Materials (iChEM), Dalian Institute of Chemical Physics, Chinese Academy of Sciences, Dalian 116023, China; National Center for Magnetic Resonance in Wuhan, State Key Laboratory of Magnetic Resonance and Atomic and Molecular Physics, Key Laboratory of Magnetic Resonance in Biological Systems, Wuhan Institute of Physics and Mathematics, Innovation Academy for Precision Measurement Science and Technology, Chinese Academy of Sciences, Wuhan 430071, China; National Center for Magnetic Resonance in Wuhan, State Key Laboratory of Magnetic Resonance and Atomic and Molecular Physics, Key Laboratory of Magnetic Resonance in Biological Systems, Wuhan Institute of Physics and Mathematics, Innovation Academy for Precision Measurement Science and Technology, Chinese Academy of Sciences, Wuhan 430071, China; Key Laboratory of Separation Science for Analytical Chemistry, Dalian Institute of Chemical Physics, Chinese Academy of Sciences, Dalian 116023, China; Energy College, University of Chinese Academy of Sciences, Beijing 100049, China; National Engineering Laboratory for Methanol to Olefins, Dalian National Laboratory for Clean Energy, Collaborative Innovation Center of Chemistry for Energy Materials (iChEM), Dalian Institute of Chemical Physics, Chinese Academy of Sciences, Dalian 116023, China; Key Laboratory of Separation Science for Analytical Chemistry, Dalian Institute of Chemical Physics, Chinese Academy of Sciences, Dalian 116023, China; National Center for Magnetic Resonance in Wuhan, State Key Laboratory of Magnetic Resonance and Atomic and Molecular Physics, Key Laboratory of Magnetic Resonance in Biological Systems, Wuhan Institute of Physics and Mathematics, Innovation Academy for Precision Measurement Science and Technology, Chinese Academy of Sciences, Wuhan 430071, China; National Engineering Laboratory for Methanol to Olefins, Dalian National Laboratory for Clean Energy, Collaborative Innovation Center of Chemistry for Energy Materials (iChEM), Dalian Institute of Chemical Physics, Chinese Academy of Sciences, Dalian 116023, China; National Engineering Laboratory for Methanol to Olefins, Dalian National Laboratory for Clean Energy, Collaborative Innovation Center of Chemistry for Energy Materials (iChEM), Dalian Institute of Chemical Physics, Chinese Academy of Sciences, Dalian 116023, China; State Key Laboratory of Catalysis, Dalian Institute of Chemical Physics, Chinese Academy of Sciences, Dalian 116023, China; Energy College, University of Chinese Academy of Sciences, Beijing 100049, China

**Keywords:** zeolite, MTO reaction, diffusion, shape-selective catalysis, multiscale dynamical cross-talk

## Abstract

Establishing a comprehensive understanding of the dynamical multiscale diffusion and reaction process is crucial for zeolite shape-selective catalysis and is urgently demanded in academia and industry. So far, diffusion and reaction for methanol and dimethyl ether (DME) conversions have usually been studied separately and focused on a single scale. Herein, we decipher the dynamical molecular diffusion and reaction process for methanol and DME conversions within the zeolite material evolving with time, at multiple scales, from the scale of molecules to single catalyst crystal and catalyst ensemble. Microscopic intracrystalline diffusivity is successfully decoupled from the macroscopic experiments and verified by molecular dynamics simulation. Spatiotemporal analyses of the confined carbonaceous species allow us to track the migratory reaction fronts in a single catalyst crystal and the catalyst ensemble. The constrained diffusion of DME relative to methanol alleviates the high local chemical potential of the reactant by attenuating its local enrichment, enhancing the utilization efficiency of the inner active sites of the catalyst crystal. In this context, the dynamical cross-talk behaviors of material, diffusion and reaction occurring at multiple scales is uncovered. Zeolite catalysis not only reflects the reaction characteristics of heterogeneous catalysis, but also provides enhanced, moderate or suppressed local reaction kinetics through the special catalytic micro-environment, which leads to the heterogeneity of diffusion and reaction at multiple scales, thereby realizing efficient and shape-selective catalysis.

## Teaser

The multiscale dynamical cross-talk in zeolite-catalyzed methanol and dimethyl ether conversions is unveiled, suggesting the best spatiotemporal cooperation of diffusion–reaction–material is pivotal for optimal zeolite catalysis and process application.

## INTRODUCTION

Diffusion—a universal physical phenomenon in nature affecting many physical and chemical processes at both microscopic and macroscopic scales—is of great significance for both fundamental research and industrial applications [1]. It is the basis and efficiency determinant for many practical applications for shape-selective separation and catalysis over nanoporous solid materials, especially zeolites [1–4].

Two prominent features of zeolites, molecular sieving and the confinement effect, enable shape-selective catalysis by modulating the mass transport of reactants, intermediates and products in molecule-sized confines. In particular, a representative example is the successfully industrialized methanol-to-olefins (MTO) process that is catalyzed by SAPO-34, characterized by a large CHA cavity and a small eight-membered ring (8-MR) window, which hinders the diffusion of larger hydrocarbons and selectively sieves ethene and propene as end (and desired) products [4–8]. Numerous works [5–7,9,10] have revealed the C-C bond formation mechanism of the MTO reaction and its dynamic reaction features. Several scientific explanations have been proposed for the formation of the first C-C bond, and the dynamic reaction process guided by surface organic species is being established [6,7,9]. After a rather short induction period, the MTO process shifts to the steady-state stage driven by the autocatalytic hypercycle network [9]. A typical working MTO catalyst can be described as a supramolecular micro-environment system, consisting of an inorganic CHA cavity with Brønsted acid sites (BASs) and the retained active organic species [9,11]. In such a hybrid system, olefin, methylcyclopentadiene (MCP) and aromatic species work as an (auto)catalyst: they not only independently guide their respective catalytic cycles but also operate in concert to build a hypercyclic reaction network, efficiently driving methanol conversion [9]. With the reaction going on, the active species eventually age into inactive polycyclic aromatic hydrocarbons, and the deposition of these species detrimentally induces catalyst coking and deactivation [9,12]. The dynamically evolved retained organic species associated with the complex reaction network, together with the coke deposition, endow the MTO catalyst materials with the dynamic evolution nature (that is, time-dependent material) [9], which in turn affects and induces the dynamical evolution of diffusion and reaction of molecules in zeolite and eventually mediates the product distribution [13]. Thus, shape-selective catalysis for the MTO reaction is not simply related to the narrow 8-MR window of SAPO-34, but also highly related to the resided organic species (which modify acidity and cavity) and the intricate reactions driven by them.

All three categories of shape-selective catalysis [2,14]—reactant shape selectivity, transition-state shape selectivity and product shape selectivity—are wholly embodied in the MTO reaction. Transition-state shape selectivity is modulated by the confined space of molecular sieve catalysts that affect the formation of critical intermediates, thus influencing the dominant reaction routes and the product distributions [8,10,15]. Product shape selectivity has been extensively adopted to explain product selectivity [8,10,15]. Alkene and alkane probe molecules are widely used to study product diffusion and product shape selectivity [16–18], but detailed study involving the effect of deposited coke under working MTO reaction conditions is lacking. However, reactant shape selectivity is less explored [8,14,15], especially considering the diffusion of dimethyl ether (DME), the equilibrium product of methanol.

**Scheme 1.**
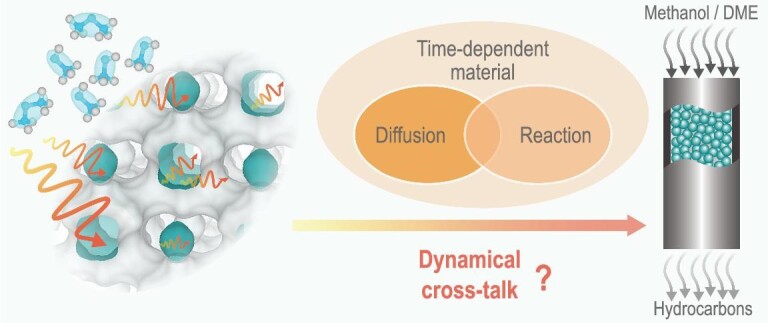
The dynamical cross-talk among time-dependent material, diffusion and reaction at multiple scales, from the molecular scale to the catalyst-crystal and catalyst-ensemble scale.

Furthermore, research on diffusion and reaction for the MTO process has often been separated. Several studies have made attempts to address diffusion and reaction simultaneously. Chen *et al*. suggested a relationship between the effective methanol and DME diffusivity and the intrinsic rate constant of MTO and DTO (DME-to-olefins) as the reaction progresses [19–21]. Weckhuysen *et al*. visualized the evolution of carbonaceous species in large single crystals of SAPO-34 during the MTO process by use of confocal fluorescence microscopy (CFM) [22–24]. Liu's group illustrated the methanol conversion process in a single SAPO-34 catalyst crystal by a feasible deep data approach [25]. Recently, Bhan *et al*. analyzed mass transport in the complex MTO process and found that diffusional hindrance significantly affects some critical steps of the MTO reaction cycles [26]. Despite this progress, there still exists a huge gap between the harvest from the experiments and the real reaction and diffusion process. This gap may originate from the heterogeneities of the heterogeneous catalyst, resulting in space- and time-dependent occurrence at multiple scales [22,27–29], and complicating the diffusion and reaction. Until now, multiple advanced spectroscopy experimental methods [22,27–29] and multiscale modeling theoretical simulation [25,30–32] have been used to understand the diffusion and reaction in zeolites at multiple scales. However, most research on diffusion and reaction for the MTO process is focused on a single scale, with experimental research being performed at the macro catalyst-ensemble scale and calculation simulation at the molecular scale. It is hard to get a full picture of the MTO mechanism when multiscale consideration of diffusion and reaction are lacking. What is more, the overlapping of dynamic autocatalysis of MTO and multiscale heterogeneities further augments the complexity of the MTO reaction system. In this context, it is challenging but imperative to unravel the dynamical interactive effect of diffusion and reaction within the time-dependent MTO zeolite materials or the catalytic micro-environments involved. This special and complicated cross-talk occurs dynamically among material, diffusion and reaction at multiple scales, ranging from the molecular scale to the catalyst-crystal scale and catalyst-ensemble scale (Scheme 1).

To tackle the aforementioned challenges, in this work we manage to disclose the dynamical multiscale diffusion and reaction process for methanol and DME conversion over SAPO-34 by integrating kinetic analysis, spatiotemporal analysis of reaction and deactivation, and theoretical simulations. Conflict between apparent and intrinsic reactivity of methanol and DME over SAPO-34 was disclosed, with reactant molecule diffusion as the origin. The mass transport property of reactant molecules, including intracrystalline diffusion and surface barriers as well as inter-cavity hopping behaviors, are well probed by uptake experiments and molecular dynamics (MD) simulations. The spatiotemporal distribution of carbonaceous species on the catalyst bed and in a single catalyst crystal are unveiled and well explained by the dynamical interplay of diffusion and reaction over the time-dependent zeolite material. The constrained diffusion of DME elongates the reaction zone of the catalyst bed, alleviates the high local chemical potential of reactants (which is the case for methanol) and thus slows down the growth of heavier coke, enabling the diffusion of DME into the inner part of the catalyst crystal and enhancing the utilization of active sites. As a very complicated catalytic system, zeolite catalysis, especially MTO catalysis, requires the deliberate optimal coordination of material, diffusion and reaction to achieve rational strategies for improved reaction efficiency and optimized shape-selective catalysis.

## RESULTS AND DISCUSSION

### Conflict between apparent and intrinsic reactivity of methanol and DME over SAPO-34 with reactant molecule diffusion as the origin

**Figure 1.**
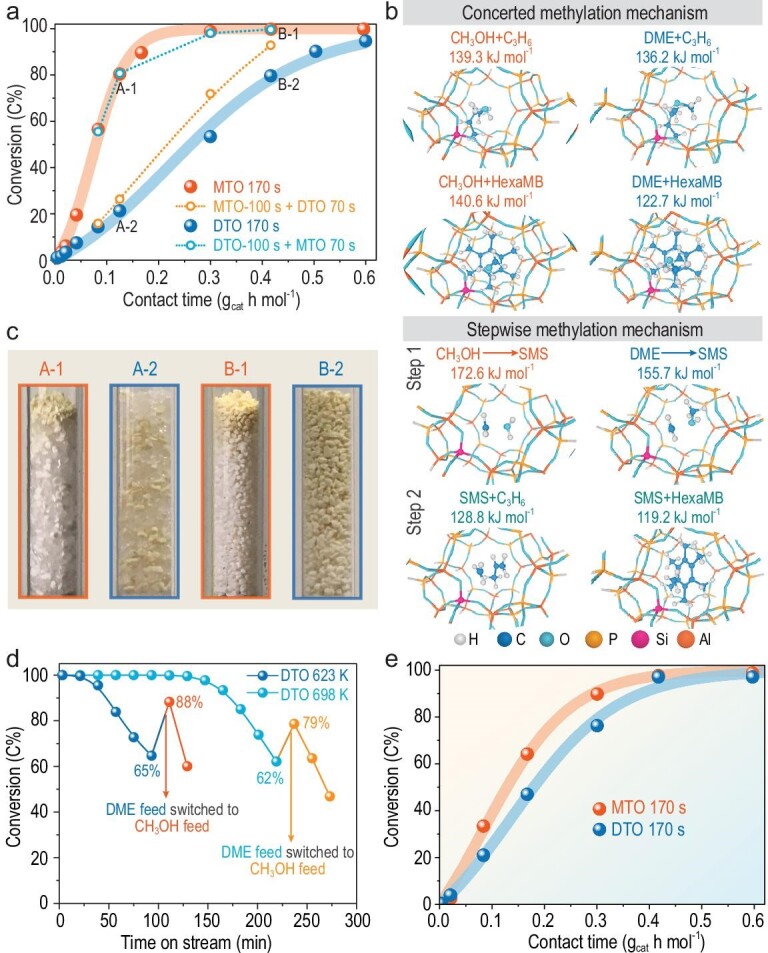
(a) Conversions of methanol and DME over SAPO-34 (8 μm) at 623 K with contact time. (b) Optimized transition state structure and free energy barrier of methylation reactions between propylene/hexaMB and methanol/DME over SAPO-34 at 623 K through the concerted and stepwise mechanisms. (c) Photographs of the catalyst bed after MTO (A-1, B-1) and DTO (A-2, B-2) reactions for 100 s, respectively, over SAPO-34 at 623 K. Reaction A-1 and A-2: W/F = 0.125 g_cat_ h mol^–1^; reaction B-1 and B-2: W/F = 0.417 g_cat_ h mol^–1^. (d) Methanol switch experiments after DME reaction. DTO reaction for 108 min at 623 K and 234 min at 698 K, respectively, followed by a switch to methanol feed. Equimolar carbon of methanol and DME was fed in (0.0062 mol_CH2_ h^–1^). (e) Conversions of methanol and DME with contact time after a 170 s reaction over SAPO-34–1 μm at 623 K.

Figure 1a displays the apparent reactivity profiles of MTO and DTO reactions performed over SAPO-34 (8 μm) (Fig. S1 in the online supplementary file) at 623 K. Prior to the full conversion, a higher reactivity of methanol was present compared to DME. This observation was in accordance with the previous results performed over SAPO-34 [33–35], but was contrary to the HZSM-5-catalyzed reactions reported in our recent work [9] and other works [36–39], where the reactivity of methanol was, instead, lower than that of DME. The reactivity of these C1 species is an important (as they act as initiators for constructing the initial C-C bond [9]) and yet controversial issue. One wonders, first, if such apparent reactivity differences are derived from the product water effect, given that product water release is different for methanol and DME conversion. However, although the DTO reaction produces less water than the MTO reaction, this is not the cause of the low reactivity of DME, as less co-fed water will exert a marginal effect on the DTO reaction and excessive co-fed water will lower the DME reactivity [9].

Such a controversial issue urges us to examine the intrinsic reactivity of methanol and DME over SAPO-34 with the help of theoretical calculation. After the induction period at the very beginning of the reaction, the autocatalytic reaction (indirect mechanism) dominates the transformation of the reactant. As shown in Scheme S1, the chemical nature of the indirect mechanism for methanol conversion can be simply described as methylation of the active intermediates followed by cracking or elimination to generate light olefins [40,41]. For the indirect mechanism of MTO and DTO reactions (Scheme S1), they share the same cracking or elimination steps, and hence the activity differences may originate from the difference in methylation activity for methanol and DME. In view of this, we conducted density functional theory (DFT) theoretical calculations to study the intrinsic reactivity of methanol and DME toward alkenes (represented by propylene) and arenes (represented by hexamethylbenzene (hexaMB)) over SAPO-34. The concerted and stepwise mechanism are both considered. As shown in Fig. 1b, for the concerted mechanism, the free energy barriers for DME methylation were lower than that for methanol, especially for the hexaMB (lower by 18 kJ/mol). For the stepwise mechanism, the free energy barrier for the formation of the surface methoxy species (SMS) for DME was also lower than that for methanol. It follows that DME is, in essence, more reactive than methanol over SAPO-34 from the crucial elementary reaction step views, the same as the conclusions reached for HZSM-5 [9]. Consequentially, the question arises as to what the cause is for the higher apparent activity of MTO than DTO over SAPO-34, which conflicts with their intrinsic activity.

To rule out the possible effect arising from the differences in chemical micro-environment of the working material of SAPO-34 during the MTO and DTO reactions, we carried out MTO and DTO reactions over methanol/DME pre-reacted SAPO-34 with an identical working micro-environment. Taking the methanol pre-reaction as an example, a methanol pre-reaction was performed for 100 s, followed by feeding methanol or DME for 70 s to distinguish their reactivity in the same catalytic micro-environments. The results clearly show that whether the catalytic material is modified by a methanol or DME pre-reaction, the apparent MTO reactivity was always higher than that of DTO (Fig. 1a).

**Figure 2.**
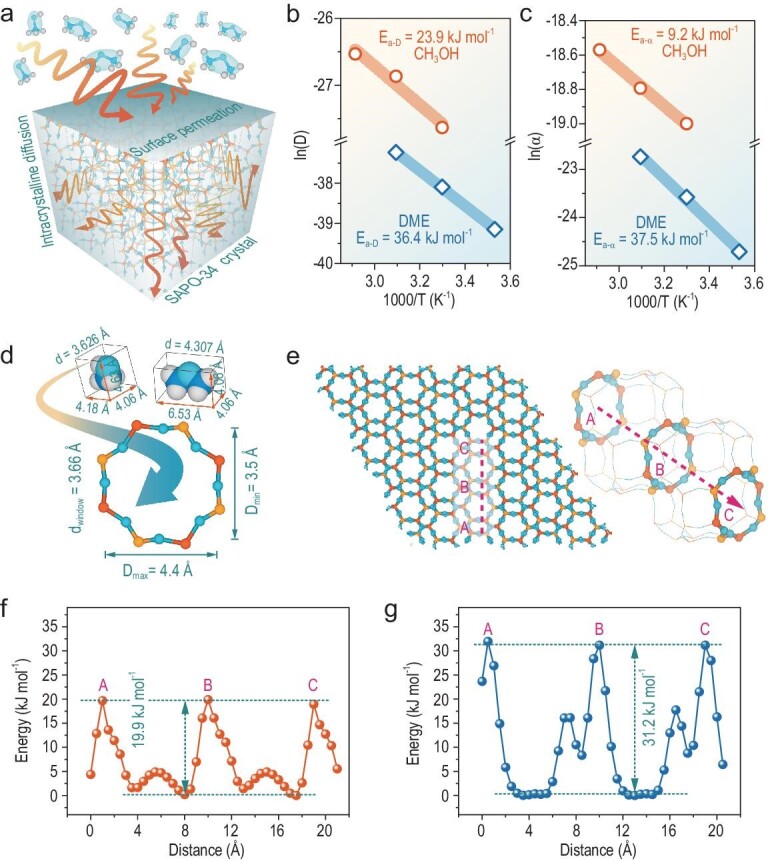
Diffusion behavior of methanol and DME over SAPO-34. (a) Scheme for diffusion behavior of methanol and DME over SAPO-34 crystal. (b and c) Arrhenius plots of (b) intracrystalline diffusivity and (c) surface permeability. (d) The molecular kinetic diameter d [44] as well as molecular dimension and configuration of methanol and DME; the characteristic parameters of SAPO-34: the minimal (D_min_) and maximal (D_max_) window size of 8-MR in a CHA cavity, according to the results of an *ab initio* molecular dynamics simulation [18]; the window diameter d_window_ described by the diameter of the largest-free-sphere that can diffuse through the CHA framework with pure SiO_2_ composition [45]. (e) The defined diffusion pathways. A molecule diffuses from the center of one cavity to the adjacent one through the 8-MR window. (f and g) Interaction energy profiles of (f) methanol and (g) DME as a function of the distance at 623 K, with a loading of one molecule per cavity, where a methanol or DME molecule diffuses from cavity A to the adjacent cavity C.

The above analyses imply that the reason for the higher apparent reactivity of MTO over SAPO-34 does not lie in the molecularly intrinsic reactivity or microscopically chemical environments. Next, we turn to find clues on the catalyst-bed scale. Two groups of photographs of the catalyst bed after MTO (A-1, B-1) and DTO (A-2, B-2) reactions (Fig. 1a) for 100 s, respectively, over SAPO-34 at 623 K, are shown in Fig. 1c, with the similar retained hydrocarbon species analyzed by gas chromatograph-mass spectrometry (GC-MS) spectra as shown in Fig. S2. Unexpectedly, the utilization degree of the catalyst bed after two reactions diverged greatly. After the MTO reaction, only the top layer of the catalyst bed turned light yellow (A-1 and B-1 in Fig. 1c), meaning hydrocarbon deposition, while for the DTO reaction, almost the entire catalyst bed turned light yellow with hydrocarbons deposited (A-2 and B-2 in Fig. 1c). Considering the larger molecular size of DME relative to methanol (Fig. 2c), we reason that, presumably, DME will be subjected to a relatively strong diffusion limitation over SAPO-34, thus resulting in the difficulty in diffusing into SAPO-34 zeolite. Such behavior would bring about the lower apparent reactivity of DTO relative to MTO and the extension of reaction zones for the DTO reaction.

To verify this deduction, methanol switch experiments after the DTO reaction were performed. When conversions of DTO reactions descended to ∼65% at the two separate temperatures of 623 and 698 K, the feed was switched to methanol, with conversions ascending to 79% and 88% (Fig. 1d). This phenomenon implies that high methanol conversion can still be achieved over the catalyst in which DME reactivity declines apparently, indicative of the constrained reactant diffusion of DME. Moreover, we conducted the control experiments over SAPO-34 with a small crystal size, as the small crystal size will reduce the diffusion hindrance [19,21,34]. On SAPO-34–1 μm, the apparent reactivity of the DTO reaction can parallel MTO (Fig. 1e), unlike that on SAPO-34 (8 μm) where the apparent reactivity of the DTO reaction was much lower than that of MTO (Fig. 1a). These results stand as proofs of the strong mass transport limitation for DME relative to methanol occurring over SAPO-34, which masks the exertion of the higher intrinsic reactivity of DME, thus showing lower apparent DTO reactivity than that of MTO, well explaining the conflict between apparent and intrinsic reactivity of methanol and DME over SAPO-34. The above analyses show that the mass transfer of the reactant affects the apparent reactivity of heterogeneous catalytic reactions. Thus, before detailing the dynamical multiscale diffusion and reaction process for MTO and DTO, the diffusion behavior of reactants should be well studied first.

### Diffusion behaviors of methanol and DME on the external surface and in the intracrystalline space of SAPO-34

The diffusion of reactants, enabling reactants to arrive at the active sites of the catalyst, is the prerequisite and basis for chemical transformations in heterogeneous catalysis [1,22,28,29]. For diffusion in nanoporous crystalline materials (e.g. SAPO-34), reactant molecules need to overcome at least two mass transfer resistances: the barriers for the permeation through the external surface (surface barriers) and for the transport in the intracrystalline confined space of the catalyst (Fig. 2a) [1,28,29]. In order to directly disclose the diffusion behaviors of methanol and DME over SAPO-34, uptake experiments from the macroscopic scale combined with theoretical MD calculations from the microscopic scale were conducted.

Based on the uptake experiments under sufficiently low molecular loading at different temperatures conducted by an intelligent gravimetric analyzer (IGA) (Fig. S3), intracrystalline (transport) diffusivity (*D*) and surface permeability (α) were successfully decoupled from the ensemble experimental measurements by the method [42] described in Supplementary Note 1. The α of methanol and DME was quantified by fitting the initial uptake rate (Fig. S3b and d) with Supplementary Equation (3). Subsequently, based on α, *D* was yielded by fitting all uptake rate data (Fig. S3a and c) with Supplementary Equation (5). With *D* and α at different temperatures (Table S1), the activation energies of intracrystalline diffusivity (E_a-D_) and surface permeability (E_a-α_) were derived by the Arrhenius Law (Supplementary Equations (6) and (7)), with the results presented in Fig. 2b and c. At 303 K, the calculated *D* of methanol was 1.00 × 10^–12^ m^2^/s, which was consistent with the intracrystalline self-diffusivity measured by pulsed field gradient nuclear magnetic resonance (PFG NMR) [42], and the data measured by the microscopical method, such as interference microscopy (IFM) [29]. The *D* of DME at 303 K was 2.85 × 10^–17^ m^2^/s, which was comparable with that of propane (with a similar kinetic diameter of 4.3 Å [43]) measured by IGA at 313 K (1.16 × 10^–16^ m^2^/s) [42], and was five orders of magnitude smaller than that of methanol. Accordingly, both E_a-D_ and E_a-α_ for DME (36.4 and 37.5 kJ/mol, respectively) were higher than that of methanol (23.9 and 9.2 kJ/mol, respectively). The differences between such molecular diffusion behaviors are conceptually ascribed to the relatively bulky size of DME compared to methanol (e.g. the larger molecular kinetic diameter of DME (4.307 Å) than methanol (3.626 Å) [44], and the parameters of 8-MR in CHA cavity [18,45] are shown in Fig. 2d), which is the origin of the reactant shape selectivity.

Subsequently, MD simulation at a reaction temperature of 623 K was employed to arrive at a microscopic interpretation of the diffusion behavior of methanol and DME in SAPO-34 at the molecular level. The diffusion behavior of a guest molecule in cavity-type molecular sieves, such as SAPO-34, is an inter-cavity hopping event where the guest molecule jumps from one energy minimum to another by overcoming energy maximum, i.e. jumping between adjacent cavities through 8-MR [2,16,17,46]. The energy minimum is located in the spacious cavity, while the narrow 8-MR, imposing very strong steric hindrance for the inter-cavity hopping, is the energy maximum [16,17,46]. To quantitatively assess such an inter-cavity hopping event, the interaction energies (representing the magnitude of the host–guest interactions between host molecular sieves and guest molecules along the diffusion path) [47] associated with the energy barrier defined by the 8-MR crossing obstacle, were adopted. The corresponding interaction energy profiles and the determined energy barriers for methanol and DME along the defined diffusion path (Fig. 2e) at 623 K are shown in Fig. 2f and g, respectively. The energy barrier for DME crossing 8-MR (31.2 kJ/mol) was higher than that for methanol (19.9 kJ/mol). These two theoretically calculated barriers are comparable with the experimentally determined E_a-D_ of 36.4 kJ/mol for DME and 23.9 kJ/mol for methanol (Fig. 2b). Theoretically, the inter-cavity hopping ability is determined by jump frequency (or hopping time, the average waiting time of a guest molecule within a CHA cavity for each jump) and jump length (the average distance between two CHA cavities, 9.1 Å) [46]. Compared with methanol, the higher barrier for DME crossing the 8-MR window will hinder its inter-cavity motions and will then lead to the lower jump frequency, thus generating limited three-dimensional walking distances inside the SAPO-34 crystal within an identical diffusion time.

Originating from the nature of the reactant molecules, especially the difference in molecular size, methanol and DME molecules exhibit different diffusion behaviors. Both methanol and DME could diffuse into a SAPO-34 crystal. However, compared with methanol, it is relatively difficult for DME, with its bulky size, to permeate the external surface of SAPO-34 due to its lower surface permeability, and DME also needs to overcome a higher energy barrier for crossing 8-MR during intracrystalline diffusion, consequently showing depressed mass transfer properties. These diffusion behaviors of methanol and DME would create a diverse diffusion and reaction process at multiple scales, leading to various types of shape-selective catalysis.

### The spatiotemporal distribution of carbonaceous species on a catalyst bed

The zeolite-catalyzed MTO/DTO reaction is a special diffusion and reaction process for heterogeneous catalysis, where along with the generation of gas-phase effluent products, large carbonaceous species are gradually formed and confined in the solid-phase catalyst as retained products (coke) to dynamically modify the catalyst material. Such time-dependent material, in turn, affects the dynamical diffusion and reaction process of the MTO/DTO reaction proceeding in it. These processes manifest the special cross-talk among the catalyst material, diffusion and reaction.

**Figure 3.**
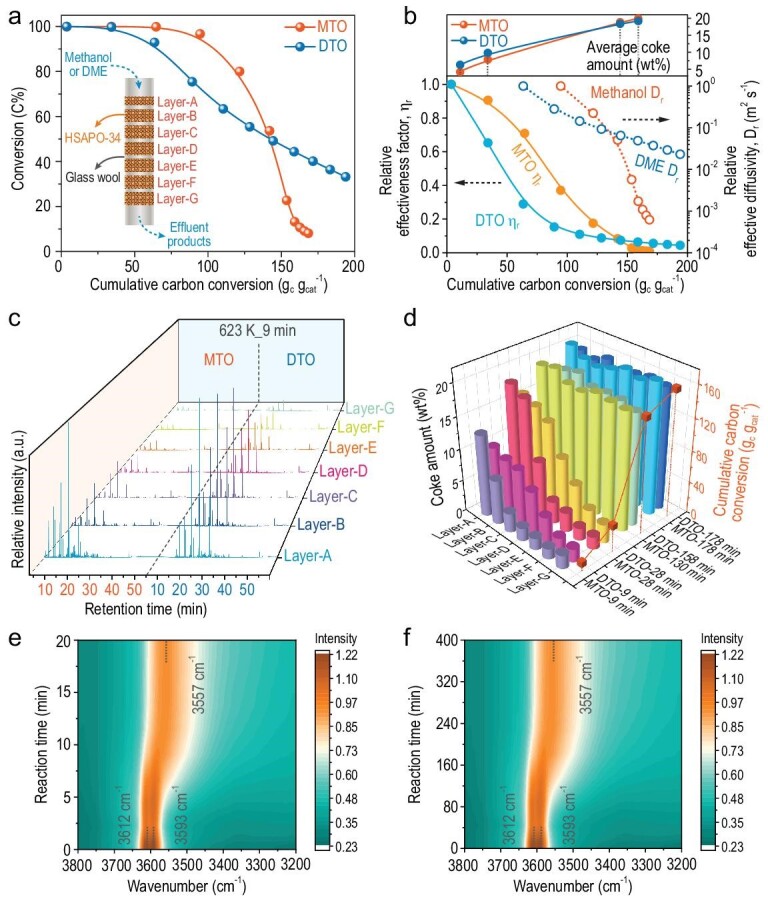
(a) Conversions with cumulative carbon conversion for MTO and DTO reactions over SAPO-34 at 623 K, with equimolar carbon of methanol and DME feeding (0.0062 mol_CH2_ h^–1^). The corresponding product distributions are shown in Fig. S4. The inset illustration is the fixed-bed reactor with catalyst loaded in seven layers. (b) Relative effectiveness factor (η_r_), relative effective diffusivity (D_r_) of methanol and DME, and average coke amount of the entire catalyst bed with cumulative carbon conversion for MTO and DTO reactions, respectively, over SAPO-34 at 623 K. The initial value of η_r_ is set to 1, and it should be noted that only when the value of η_r_ is lower than 0.37, D_r_ is calculated and set to 1 as the initial value. The calculation details are shown in Supplementary Note 2. (c) GC-MS chromatograms of confined soluble carbonaceous species confined in a seven-layer catalyst bed (Layer-A to Layer-G) after methanol and DME conversion at 623 K for 9 min; the results after reaction for 28 and 178 min are shown in Fig. S5. All peaks are normalized relative to the internal standard peak C_2_Cl_6_, indicated by * in the chromatograms. (d) The amount of coke (total amount of confined carbonaceous species) formed in the seven-layer catalyst bed after methanol and DME conversion for various amounts of time. (e and f) The remaining BASs monitored by *in situ* DRIFT spectra during (e) MTO and (f) DTO reactions over SAPO-34 at 623 K.

The cross-talk involving the dynamical diffusion and reaction process for MTO and DTO reactions over SAPO-34, under real reaction conditions, is shown in Fig. 3a and b. With cumulative carbon conversion, the catalyst was gradually modified by carbonaceous species, and the average coke amount of the entire catalyst bed was gradually increased. Concurrently, the relative catalyst effectiveness factor (η_r_) and relative effective diffusivity (D_r_) of methanol and DME dropped rapidly, with a higher decrease rate for η_r_. Notably, DME experienced earlier catalyst-bed penetration and yet the subsequent reactivity, as well as the η_r_ and D_r_, declined slowly relative to methanol. The product water effect (considering the lower water concentration in DTO compared to MTO) on such cross-talk differences has been ruled out by DME-H_2_O co-feeding experiments (Fig. S6), in which the co-fed water has less effect on the reaction features of the DTO reaction. Next, we elucidate the dynamic multiscale cross-talk behaviors and mechanisms among time-dependent material, diffusion and reaction in MTO and DTO reactions by the spatiotemporal distribution of carbonaceous species at multiple scales.

Firstly, the occurrence of the cross-talk at the scale of the catalyst bed was elucidated by the dynamic evolution of the reaction zone and the deactivation pattern. To reveal the spatial distribution of carbonaceous species, the catalyst bed along the axis of the fixed-bed reactor was divided into seven layers (Fig. 3a). GC-MS analyses of soluble carbonaceous species (‘soluble coke’) confined in different catalyst bed layers after MTO and DTO reactions for 9 min (Fig. 3c), as well as 28 and 178 min (Fig. S5), show that the chemical nature of the confined soluble carbonaceous species (Fig. S7) was identical for both reactions and independent of the position in the catalyst bed, mainly including abundant methylbenzene and methylnaphthalene, and slight methyladamantane, phenanthrene and pyrene. However, the spatiotemporal distribution of the confined carbonaceous species in the seven layers of the catalyst bed, for two reactions, differed largely (total (coke) amount is shown in Fig. 3d and Fig. S8; ‘soluble coke’ amount is shown in Fig. 3c and Fig. S6). At the early reaction stage (9 min), for the MTO reaction, the occluded carbonaceous species were mainly localized on the top layer of the catalyst bed (coke amount, 12.9%) and the amount was sharply decreased from the second layer. However, the case was quite different for the DTO reaction, where the coke amount in the top layer (10.2%) was lower than that of the MTO reaction, but the main coke distribution spectrum was broad—gradually stepping down from the top layer to the fifth layer. With the reaction proceeding to 28 min, the coke amount increased for all layers of two reactions, but the increment was mainly concentrated in the top few layers. Even so, the coke distribution spectrum for the MTO reaction was getting broader than that at 9 min, and its axial gradient difference of coke relative to the DTO reaction was noticeably reduced. Such an axial gradient difference in coke amount for the MTO and DTO reaction was further eliminated as the reaction progressed and became marginal after 178 min, where the coke amounts of the seven catalyst bed layers for the MTO and DTO reaction were comparable.

Of particular note is that, after 178 min of reaction, although MTO conversion had declined to 13%, DTO conversion was, unexpectedly, still as high as 44%. This observation is surprising, in view of the fact that after 178 min, the chemical nature of confined organic species (Fig. S7), and the coke amounts for each layer of the catalyst bed, as well as cumulative carbon conversion (Fig. 3d and Fig. S8) for both the MTO and DTO reactions, were all comparable. These phenomena allow us to reasonably speculate that the acidity utilization of catalyst material and the spatial distribution of carbonaceous species in a single SAPO-34 catalyst crystal for MTO and DTO reactions might be different.

The acidity utilization during the conversion of methanol and DME was quantitatively monitored by *in situ* diffuse reflection infrared Fourier transformations spectroscopy (DRIFTS). Only the surface layer of the catalyst in the *in situ* cell was monitored in the *in situ* DRIFTS experiments, which is similar to the topmost layer of the catalyst bed in a fixed-bed reactor. As shown in Fig. 3e and f, as the reaction progresses, the bands at 3612 and 3593 cm^–1^, attributing to two types of bridging hydroxyl groups (that is, BASs of SAPO-34) [48], gradually disappear upon the interaction of BASs with adsorbate molecules (reactants and hydrocarbon products). Concurrently, a broad band centered at 3557 cm^–1^ appeared, attributed to the Si(OH)Al groups interacting with the confined carbonaceous species as adsorbates. BASs of SAPO-34 for the DTO reaction were consumed slower than that for the MTO reaction, indicating the quick MTO reaction and intensified carbonaceous deposition in the local catalyst micro-environment in the MTO reaction. Therefore, when the reaction proceeds to the same stage, abundant BASs still remained in the catalyst of the DTO reaction, but the catalyst of MTO encountered the quick loss of BASs in large amounts.

### The spatiotemporal distribution of carbonaceous species in a single catalyst crystal

Next, the advanced super-resolution structured illumination microscopy (SIM) measurement was performed to directly visualize the dynamically spatiotemporal evolution of carbonaceous species in a single catalyst crystal during the MTO and DTO reactions. The previous *in situ* ultraviolet-visible (UV-vis) experiments [23,24,49,50] and time-dependent DFT calculations [24,25,50] confirmed that the characteristic UV-vis absorption bands of benzene-, naphthalene-, phenanthrene- and pyrene-based carbenium ions with methyl substituents are around 390, 480, 560 and 640 nm, respectively, and the corresponding emission wavelengths are located in the range of 480–490, 500–520, 620–630 and 670–700 nm, respectively. The characteristic excitation and emission wavelengths of the aforementioned carbenium ions are fully covered by the SIM measurements in this work.

**Figure 4.**
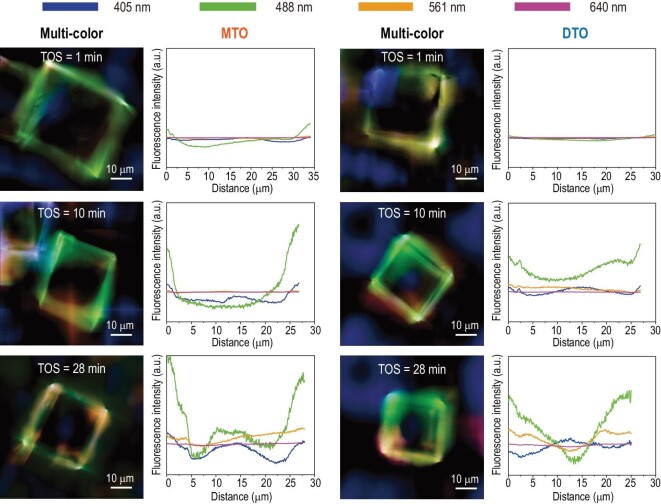
The spatiotemporal evolution of carbonaceous species obtained by SIM over a single SAPO-34-30 μm crystal during MTO and DTO reactions, respectively, with time on stream at 623 K. The SIM images are the fluorescence originating from the overlap of four profiles with different laser excitations of 405 nm (detected at 435–485 nm, false color: blue), 488 nm (detected at 500–545 nm, false color: green), 561 nm (detected at 570–640 nm, false color: yellow) and 640 nm (detected at 663–738 nm, false color: pink). The images were taken in the middle plane of the zeolite crystal.

As shown in Fig. 4, as the MTO reaction progresses, the carbonaceous species, which lights up the SAPO-34 crystal, start to form in the crystal rim and then gradually expand to the interior of the crystal. Concurrently, active carbonaceous species (benzene-based carbenium ions) were gradually transformed into polycyclic coke species in the crystal rim, which can block the micropore and decrease the accessibility of the inner acid sites to the reactant, eventually causing catalyst deactivation. A similar spatiotemporal evolution of carbonaceous species occurred for the DTO reaction. However, the light-emitting region (the crystal rim where the carbonaceous species were located) for the DTO reaction extended more deeply into the crystal than that for the MTO reaction after 28 min of reaction. These results show that due to the consequence of diffusion divergence for methanol and DME over SAPO-34, MTO and DTO reactions present drastically distinct reactivity and deactivation patterns with discriminating spatiotemporal evolution and distribution of carbonaceous species, not only on the catalyst-bed scale but also on the catalyst-crystal scale.

**Scheme 2.**
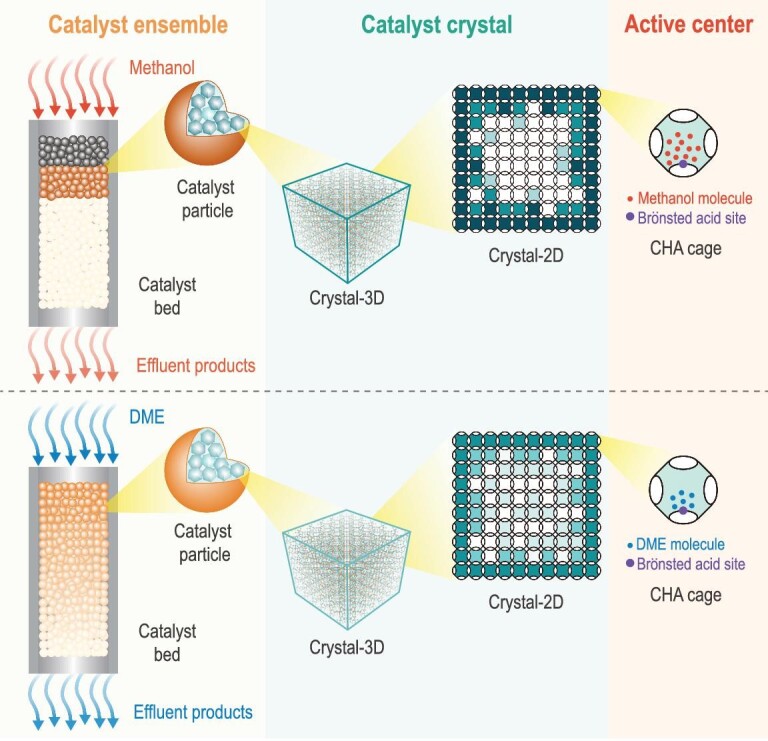
Multiscale reaction and deactivation model of MTO and DTO reactions over SAPO-34, from the catalyst-bed scale to the catalyst-crystal and CHA-cavity scale.

The above results allow us to formulate the dynamical reaction and deactivation model of MTO and DTO reactions by multiscale analysis (Scheme 2). For the MTO reaction, the reactive zone of the catalyst bed is narrow (Figs 1c, and 3c and d), with only a small number of catalysts on the top layer converting all methanol into hydrocarbons at the initial reaction stage. Thus, the local concentration of methanol molecules and the corresponding chemical potential in each CHA cavity of SAPO-34 in the reactive zone would be higher (Scheme 2). The locally enhanced chemical potential of methanol would lead to the quick MTO reaction, with the hydrocarbon species generated and further converted into the heavier aromatic or even cage-passing aromatics [12] in the rim of the catalyst crystal in the top layer of the catalyst bed. This hinders the further diffusion of the reactant into the inner part of the SAPO-34 crystal, thereby leading to the rapid deactivation of the top layer of the catalyst bed. Whereafter, the reaction zone migrates downward. Hence, the catalyst bed exhibits a layer-by-layer inhomogeneous deactivation mode (Scheme 2), which is in line with the classic ‘cigar burn’ model [11]. Accordingly, the reactivity and relative diffusivity of methanol, as well as the relative catalyst effectiveness factor of the MTO reaction, decline rapidly during the MTO reaction (Fig. 3a and b).

For the DTO reaction, due to the serious surface and intracrystalline diffusion resistance of DME over SAPO-34, a longer reactive zone of the catalyst bed is necessitated to fully convert DME (Figs 1c and 3c and d), and relatively homogenous deactivation behavior over the entire catalyst bed appears (Scheme 2). When such the longer reaction zone moves downward as the reaction progresses, the breakthrough of DME occurs earlier (Fig. 3a). However, after breakthrough, the decay of the DME reactivity and relative diffusivity, and the relative catalyst effectiveness factor of the DTO reaction is slower compared to methanol conversion (Fig. 3a and b), which can be explained on the scale of the CHA cavity and catalyst crystal. Due to the constrained diffusion of DME over the SAPO-34 crystal and the elongated reaction zone, the local molecular concentration as well as chemical potential of DME in each CHA cavity of SAPO-34 in the reactive zone would be relatively lower than that of methanol (Scheme 2). This would moderate the DTO reaction and generate less heavier coke relative to methanol conversion, as directly evidenced by the higher exothermic temperature for the spent catalyst of MTO than DTO based on temperature programmed oxidation (TPO) experiments (Fig. S9). The lighter coke deposition for DME conversion, associated with the much higher DME conversion compared to methanol during the deactivating stage and the slower BAS consuming rate (Fig. 3f), suggests that quite a part of DME can still diffuse into the inner part of the catalyst crystal to further react at the late reaction stage (Scheme 2), as directly evidenced by the SIM measurements in Fig. 4. Consequently, the reactivity and relative diffusivity of DME, and the relative catalyst effectiveness factor of the DTO reaction decline slowly (Fig. 3a and b), attaining the high reaction capacity and catalyst utilization efficiency.

Even being performed in the same zeolite material and possessing very close hydrocarbon pool mechanisms, the dynamical proceeding of the DTO reaction (relative to the MTO reaction) is distinctly regulated by the cross-talk of material, diffusion and reaction. On one side, due to the confined-organics modification, cavity type SAPO-34 material is dynamically evolved in time with the autocatalytic MTO reaction running from initiation to decay. On the other side, the time-dependent material, in turn, creates the catalysis with the dynamical evolution of diffusion and reaction. Such dynamical cross-talk among time-dependent material, diffusion and reaction occurs from the catalyst-bed scale to the catalyst-crystal and CHA-cavity scale, and eventually results in the spatiotemporal heterogeneity in carbonaceous species distribution at multiple scales, revealing the root of heterogeneous catalytic efficiency, shape-selective catalysis, especially reactant shape-selective catalysis, and deactivation mode. Originating from the reactant shape selectivity of zeolite materials, the local chemical potential of reactant molecules is different with the zeolite material usage and specific catalytic application, which progressively bring about a diverse dynamical cross-talk process at multiple scales.

## CONCLUSION

By multiscale analysis, with the aid of multi-technique approaches, this work uncovers the dynamic cross-talk behaviors and mechanisms among time-dependent material, diffusion and reaction, taking MTO and DTO reactions over SAPO-34 as prototypical reactions. Compared with methanol, mass transfer of DME is constrained over SAPO-34, since its external surface permeation and inter-cavity hopping are hindered due to the higher energy barrier of surface permeability and intracrystalline diffusivity. Such depressed diffusion masks the higher molecularly intrinsic reactivity of DME, which is independent of catalyst topology, thus leading to the lower apparent DME reactivity over SAPO-34.

The diffusion behavior of reactant molecules imposes prominent effects not only on its apparent reactivity, but also on the dynamically spatiotemporal heterogeneity of reaction and deactivation, in a multiscale manner from the CHA-cage or molecular scale to the catalyst-crystal and catalyst-bed scale. The constrained mass transfer of DME elongates the reaction zone of DTO over the catalyst bed, but also engenders the lower local chemical potential of the reactant, thereby generating moderate reaction kinetic and less heavier coke in the local catalyst micro-environment. Such cross-talk of diffusion–reaction–material (coke) occurring at microscale in the CHA cavity triggers cross-talk behaviors at multiple scales: (i) enabling quite a part of DME still to diffuse into the inner part of the catalyst crystal to further react at the late reaction stage, sustaining the turnover of DME with high capacity, and (ii) eventually leading to a relatively moderate and homogenous reaction and deactivation mode, and higher catalyst utilization efficiency. In contrast, methanol conversion exhibits a layer-by-layer inhomogeneous reaction and deactivation mode, and meanwhile, the higher local chemical potential of methanol enables the intensified reaction and deactivation localized in the outer part of the catalyst crystal, working as the main efficient zone. The multiscale cross-talk behaviors and mechanisms originate from the reactant shape selectivity of zeolite materials in the dynamical reaction procedure of MTO and DTO, and these comprehensive analyses will enrich the shape-selective catalysis.

Commercialized DMTO^®^ technology [6] perfectly employs the fluidized-bed continuous reaction-regeneration technology to achieve a high reaction efficiency, but also, simultaneously, the excellent product shape selectivity for light olefins by the usage of diffusion limitation of SAPO-34 with carbonaceous deposition. The characteristics of the DTO reaction presented in this work, moderately evolved reaction kinetics and depressed coke deposition, will prompt the different operation of its catalytic application, implying the possibility of realizing the long-term operation of a fixed-bed DTO process. The difference in the dynamical proceeding of the DTO and MTO reaction make aware that for the specific dynamic reaction catalyzed by zeolite material, achieving the best spatiotemporal cooperation of material, diffusion and reaction is the most critical strategy for the optimal catalyst development and process application.

## Supplementary Material

nwac151_Supplemental_File
