# Supplementary material for: Multiscale dynamical cross-talk in zeolite-catalyzed methanol and dimethyl ether conversions
Source: Natl Sci Rev. 2022 Aug 4;9(9):nwac151. doi: 10.1093/nsr/nwac151 (PMC9508824; doi:10.1093/nsr/nwac151)
Supplement: nwac151_Supplemental_File [file nwac151_supplemental_file.pdf]

Supplementary Information

Multi-scale dynamical cross-talk in zeolite-catalyzed methanol and dimethyl ether conversions

Shanfan Lin[1,5], Yuchun Zhi[1], Zhiqiang Liu[2], Jiamin Yuan[2], Wenjuan Liu[3,5], Wenna Zhang[1], Zhaochao Xu[3], Anmin Zheng[2], Yingxu Wei[1*] and Zhongmin Liu[1,4,5*]

[1]National Engineering Laboratory for Methanol to Olefins, Dalian National Laboratory for Clean Energy, Collaborative Innovation Center of Chemistry for Energy Materials (iChEM), Dalian Institute of Chemical Physics, Chinese Academy of Sciences, Dalian 116023, China

[2]National Center for Magnetic Resonance in Wuhan, State Key Laboratory of Magnetic Resonance and Atomic and Molecular Physics, Key Laboratory of Magnetic Resonance in Biological Systems, Wuhan Institute of Physics and Mathematics, Innovation Academy for Precision Measurement Science and Technology, Chinese Academy of Sciences, Wuhan 430071, China

[3]Key Laboratory of Separation Science for Analytical Chemistry, Dalian Institute of Chemical Physics, Chinese Academy of Sciences, Dalian 116023, P. R. China.

[4]State Key Laboratory of Catalysis, Dalian Institute of Chemical Physics, Chinese Academy of Sciences, Dalian 116023, China

[5]University of Chinese Academy of Sciences, Beijing 100049, China

[*]**Corresponding authors**. E-mails: liuzm@dicp.ac.cn; weiyx@dicp.ac.cn

SUPPLEMENTARY METHODS

Catalyst and characterizations

SAPO-34 (Si/(Si+Al+P) = 0.085) and SAPO-34-50μm (Si/(Si+Al+P) = 0.098) were synthesized following the procedure described in our previous works: ref[1] and ref[2], respectively. SAPO-34-1μm (Si/(Si+Al+P) = 0.071) were purchased from Catalyst & Catalysis Technology Co. Ltd. of CAS. The H-form SAPO-34 were obtained by calcining the crystallized products at 873 K in air for 6 h to remove the template. Scanning electron microscope (SEM) of these simples are shown in Fig. S1.

Catalytic testing

All MTO and DTO reactions were carried out in a fixed-bed quartz tube reactor with inner diameter of 4 mm under atmospheric pressure at 623 K. The SAPO-34 catalyst powder was pressed and sieved into 40-60 mesh. Prior to reaction, the catalyst was activated under He flow at 723 K for 40 min, and then the temperature was decreased to the reaction temperature. For MTO and DTO reactions, equimolar carbon amounts of methanol and DME were fed.

Typically, 100 mg SAPO-34 was used. Methanol (>99.8%) was fed by passing nitrogen through a saturation evaporator kept at 287 K. Gaseous DME (4.5 mol% dilution with helium) was directly fed at atmospheric pressure. In order to study the axial distribution of hydrocarbon species along the catalyst bed in the fixed bed reactor, 100 mg SAPO-34 was divided into seven layers by quartz wool as shown in Fig. 3a.

Differently, activity tests were conducted at varied contact time (0.004-0.596 g_{cat} h mol^{-1}). 5−100 mg SAPO-34 catalysts were diluted with 238−0 mg quartz sand (40-60 mesh) to keep the same bed volume. CH_3OH kept at 300 K and DME (9 mol% dilution with helium) were used.

Gaseous reaction products were kept at 473 K to avoid condensation and analyzed via online gas chromatography−mass spectrometer (GC−MS, Agilent 7890B/5977A) equipped with a PoraPLOT Q capillary column and a flame ionization detector. The conversion and selectivity of MTO and DTO reactions were calculated on a CH_2 basis.

Both methanol and DME were considered as reactants in the calculation.

Coke analyses

The total coke amounts of the spent catalysts were determined by thermogravimetric analysis (TGA) performed on a TA SDTQ 600 analyzer. Typically, about 10 mg sample was loaded and heated from room temperature to 1173 K with a temperature-programmed rate of 10 K min^{-1} under an air flow of 100 ml min^{-1}. The weight loss below 523 K is ascribed to the desorption of water.

20 mg spent catalysts were dissolved in 0.4 ml 20% hydrofluoric acid (HF) solution in a Teflon vial to liberate the retained species, and then it was extracted by the addition of 0.5 ml dichloromethane (CH_2Cl_2) containing hexachloroethane (C_2Cl_6) as internal standard. The CH_2Cl_2-extracted organic phase, hereinafter called "soluble coke" (molecular weight smaller than 300 g mol^{-1}), was analyzed by GC-MS (Agilent 7890A/5975C) equipped with a HP-5 capillary column and identified by reference to the mass spectral library of NIST08.

Uptake rate measurements

The uptake rate measurements were performed on an intelligent gravimetric analyzer (IGA100, Hiden Isochema Ltd., Warrington, UK). Specially, in order to accurately and stably control a tiny pressure, a high accuracy MKS pressure transducers with small range from 0 mbar to 2 mbar was used. A sensitive microbalance (resolution of 0.1 μg) was mounted in a thermostatic chamber to remove thermal coefficients of the weighing system and provide a high stability and accuracy. About 8~50 mg SAPO-34 zeolites were loaded into the microbalance bucket, and pretreatment under a vacuum less than 10^{-6} mbar at 623 K for at least 4 h until a constant weight was achieved. After that, the temperature of the sample was adjusted to the expected temperature and was regulated within 0.1 K by the constant temperature system. Then a non-flowing steam of probe gas or vapor (DME or methanol) at a given pressure (~0.06 mbar) was introduced into the system with a carefully controlling quantity in order to ensure the isobaric and isothermal process. Meanwhile, mass change (resolution of 0.1 μg) with buoyancy corrections, system pressure and sample

temperature were recorded. Monitored the dynamic adsorption process until the equilibrium was obtained under this isobaric and isothermal conditions. The uptake curves of methanol (Semiconductor Grade, 99.9 wt%, Alfa Aesar (China) Chemicals Co., Ltd.) in SAPO-34 were recorded at 303, 323 and 343 K under 0.06 mbar. The uptake curves of DME (99.9% purity, purchased from Dalian Special Gases Co., Ltd.) in SAPO-34 were recorded at 283, 303 and 323 K under 0.06 mbar.

In situ DRIFT measurements

DRIFT spectra were collected on a Bruker Tensor 27 instrument with a diffuse reflectance infrared cell with ZnSe window and a liquid nitrogen cooled Hg-Cd-Te detector. Before the measurements, 20 mg catalysts powder was loaded in the cell and flatten the surface, then pre-treated under a He flow at 773 K for 60 min and, subsequently, decreased to the reaction temperature of 623 K. CH_3OH was fed by passing 25 ml min^{-1} nitrogen through a saturator evaporator kept at 287 K. DME (4.5 mol% dilution with helium) at 27.5 ml min^{-1} was fed at atmospheric pressure to give the same CH_2-based WHSV as CH_3OH. Once the reactants were continuously introduced into the cell loaded with catalysts, the absorbance spectra were collected immediately by averaging 16 scans at 4 cm^{-1} resolution.

SIM

SIM was used to image the spatiotemporal distribution of the hydrocarbon and carbonaceous species confined in the spent SAPO-34 samples after MTO and DTO conversions with different TOS. The SIM studies were performed with a Nikon N-SIM super-resolution microscopy system with a motorized inverted microscopy ECLIPSE Ti2-E, a ×100/numerical aperture 1.49 oil-immersion total internal reflection fluorescence objective lens (CFI HP) and ORCA-Flash 4.0 sCMOS camera (Hamamatsu Photonics K.K.)[3,4]. The super-resolution SIM images were obtained by overlapping four fluorescence profiles, which were collected with the use of independently working 405, 488, 561, and 640 nm multi-laser light sources and the emission was detected with four photomultiplier tubes in the range 435–485, 500–545, 570–640, and 663–738 nm for the four lasers, respectively, which can cover the

characteristic area of excitation and emission wavelengths of benzene-, naphthalene-, phenanthrene-, and pyrene-based carbenium ions. Images were taken at a Z-plane of middle of zeolitic crystal. Imaging was performed under *ex situ* conditions at room temperature. The spent SAPO-34 samples were loaded on a glass-bottomed culture dish (35 mm dish with 20 mm well) and placed close to the objective lens to ensure the high resolution of image. The software NIS-Elements Ar and N-SIM Analysis were used to analyze the collected images and computationally reconstruct the super-resolution image.

Theoretical calculations

DFT calculations performed with the Gaussian 09 package[5] were applied to calculate the reaction energy barriers of methylation reactions. An extended 74T($SiP_{36}Al_{37}O_{119}H_{59}$) cluster model extracted from the crystallographic CHA structure (from the International Zeolite Association) was used to represent SAPO-34 and the locations of BAS were chosen at the 8-MR window, accessible for adsorbents and surrounded by maximum reaction space.[6] The ωB97XD hybrid density function with 6-31G (d, p) basis sets and semi-empirical AM1 were employed to predict the geometries of various adsorption structures and transition states. ωB97XD, a hybrid meta method, can describe empirical dispersion and long-range dispersion interactions.[7] During the structure optimizations, the 8-MR window, $(SiO)_3$-Si-OH-Al-$(SiO)_3$ active center and the adsorbed species were optimized using the ωB97XD method in the high level layer, while the rest of atoms were set in the low-level layer with semi-empirical AM1 method. All the atoms apart from the terminal H atoms were relax during the whole structure optimization. To obtain highly accurate energies, the single point energies were calculated at the level of ωB97XD/6-31G (d, p) based on the optimized structures. The frequency calculations were performed at the same level as geometry optimizations. The transition state had only a single imaginary frequency, and the adsorbed state was located at the energy minima of the potential energy surface without imaginary frequencies. The intrinsic free energies barriers ($\Delta G^{‡}$) under the real reaction condition at 623 K for each

elementary reaction was obtained from the ωB97XD/6-31G (d, p) total electronic energies and the thermal correction from the ωB97XD/6-31G(d, p): AM1 frequency calculations. The energies reported here have been corrected for zero-point vibration energies.

For the theoretical calculation of interaction energy, AlPO-34, aluminophosphates with CHA topology, was selected to replace SAPO-34 zeolites, considering the accuracy of used forcefield. Firstly, a methanol or DME molecule was placed into the center of one cavity, and then systematically moved to another center of the adjacent cavity following the diffusion path with 41 equi-spaced steps and each step moves a distance of 0.5 Å. The interaction energy between framework and molecule at each point was calculated and the energy barrier for crossing 8-MR was determined by the difference between the lowest and highest energy along the diffusion pathway[8].

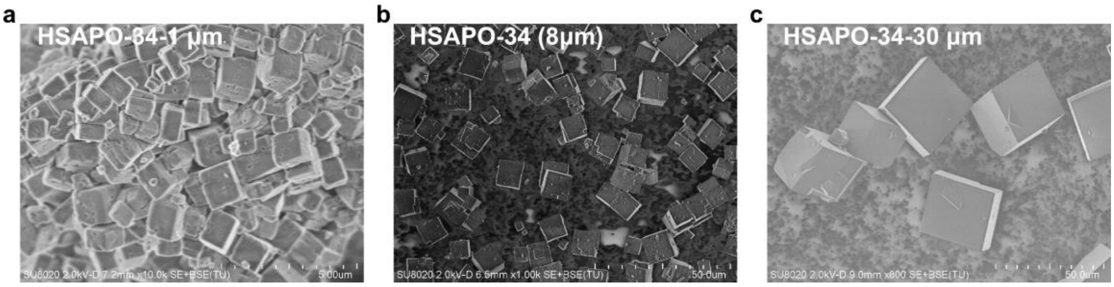

Figure S1. SEM images (characterized by a Hitachi SU8020 Cold Field Emission SEM) of SAPO-34-1 μm (a), SAPO-34 (8 μm) (b), and SAPO-34-30 μm (c) samples.

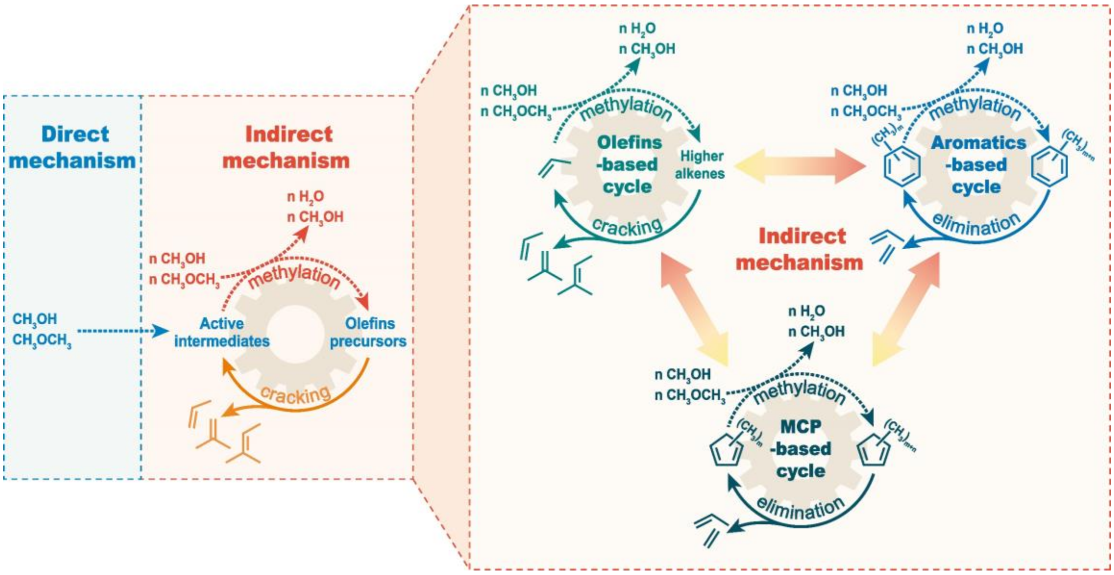

Scheme S1. The indirect mechanism of MTO and DTO reactions. The active intermediates (including olefins, methylcyclopentadiene (MCP), and aromatics), which initiates by direct mechanism, are gradually methylated with methanol/DEM to fulfill the propagation of the C-C bond, and then the extended entities (olefins precursors) split off light olefins. Olefin, MCP and aromatic species work as (auto)catalyst: they not only independently guide their respective catalytic cycles, i.e., olefins-based cycle, MCP-based cycle and aromatics-based cycle, respectively, but also operate in concert to build a hypercyclic reaction network, efficiently driving methanol and DME conversion.

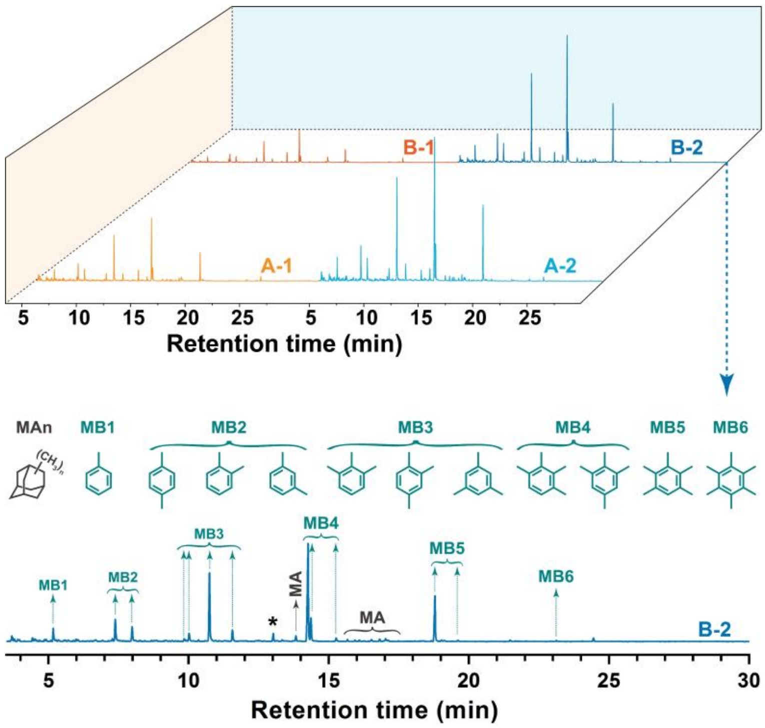

Figure S2. GC-MS spectra of the hydrocarbon species retained in SAPO-34 after MTO (A-1, B-1) and DTO (A-2, B-2) reaction for 100 s, respectively, over SAPO-34 at 623 K. All peaks are normalized relative to the internal standard peak C_2Cl_6, indicated by * in the chromatograms. The confined hydrocarbon species of the four samples are identical, mainly including abundant methylbenzene (MB) and slight methyladamantane (MA). Reaction conditions are the same as that in Fig. 1c: the contact times of reaction A-1, A-2 and B-1, B-2 are 0.125 g_{cat} h mol $^{-1}$ and 0.417 g_{cat} h mol $^{-1}$, respectively. Notably, in the case of Fig. 1c, the catalyst bed was not stratified, and for each sample, the entire bed of catalyst was used for coke analysis after homogeneous mixing. Therefore, the peak intensity of the GC-MC spectra in Fig. S2 represents the average coke amount of entire catalyst bed, which is larger for DTO (A-2, B-2) than for MTO (A-1, B-1).

Supplementary Note 1. Calculation details of intracrystalline diffusivity and surface permeability.

The relative uptake loading m_t/m_∞ of guest molecules in nanoporous materials with adsorption time can be described by the dual resistance model (DRM)[9,10], for a one-dimensional plane sheet model with a length of 2l

$$\frac{m_t}{m_\infty} = 1 - \sum_{n=1}^{\infty} \frac{2L^2 exp\left(-\frac{\beta_n^2 Dt}{l^2}\right)}{(\beta_n^2 + L^2 + L)\beta_n^2}; \beta_n \tan\beta_n = L$$

(Supplementary Equation 1)

where m_t/m_∞ is the relative uptake loading of guest molecules, t the uptake time, l the half thickness of the plane sheet, i.e., characteristic length of the intracrystalline diffusion[11], D the intracrystalline (transport) diffusivity, α the surface permeability, and $L = \alpha l/D$ the ratio of characteristic time of intracrystalline diffusion to that of surface barriers.[12] According to Fick's second law and the boundary conditions of surface mass transfer resistance, Supplementary Eq.1 can be solved by Laplace transform[10] when the initial adsorption time t is sufficiently small[12]

$$\frac{m_t}{m_\infty}\Big|^{t\to 0} = \sqrt{\frac{4Dt}{\pi l^2}} - \frac{1 - exp\left(L^2 \frac{D}{l^2} t\right) erfc\left(L\sqrt{\frac{Dt}{l^2}}\right)}{L}$$

(Supplementary Equation 2)

Expanding Supplementary Eq. (2) by Taylor series of variable $\sqrt{t}$, for sufficiently small adsorption time t, Supplementary Eq. (2) can be simplified as[12]

$$\frac{m_t}{m_\infty}\Big|^{\sqrt{t}\to 0} \cong \frac{\alpha}{l}\left(\sqrt{t}\right)^2 + O\left(\sqrt{t^3}\right)$$

(Supplementary Equation 3)

Supplementary Eq. (3) is derived based on the plane sheet, for cubic crystal, e.g. SAPO-34 zeolites used in this work, the equivalent lengths is[12]

$$l = \frac{(a/2)}{2.03}$$

(Supplementary Equation 4)

where a is the length of a cubic crystal. Supplementary Eq. (3), together with Supplementary Eq. (4) are first used to fit the measured uptake rate data by IGA at

different time interval 0 ~ $\sqrt{t}$, and a series of surface permeability and corresponding coefficient of determination of Supplementary Eq. (3) are obtained. The time interval corresponding to the maximum coefficient of determination can be used to calculate the surface permeability of cubic crystal material. With the surface permeability α determined by Supplementary Eq. (3), the intracrystalline (transport) diffusivity D can be subsequently obtained by fitting the whole uptake rate data with DRM for cubic crystal, as represented by Supplementary Eq. (5).[9,12,13]

$$\frac{m_t}{m_\infty} = 1 - \sum_{h,m,n=1}^{\infty} \frac{8L^6 exp\left(-\frac{4(\gamma_h^2+\gamma_m^2+\gamma_n^2)Dt}{a^2}\right)}{(\gamma_h^2+L^2+L)(\gamma_m^2+L^2+L)(\gamma_n^2+L^2+L)\gamma_h^2\gamma_m^2\gamma_n^2};$$

$$\gamma_i \tan\gamma_i = L = \frac{\alpha a}{2D}$$

(Supplementary Equation 5)

When L < 120, the surface permeability can be well predicted by Supplementary Eq. (3), that is the applicable range of Supplementary Eq. (3). Corresponding for L < 120, the temporal resolution for the uptake rate measurement is about ~1 s, which can be achieved by IGA apparatuses used in this work[12].

The intracrystalline diffusivity activation energies ($E_{a\text{-}D}$) were calculated by Arrhenius law:

$$D = D_0 exp(-E_{a-D}/RT)$$

(Supplementary Equation 6)

The surface permeability activation energies ($E_{a\text{-}\alpha}$) were calculated by Arrhenius law:

$$\alpha = \alpha_0 exp(-E_{a-\alpha}/RT)$$

(Supplementary Equation 7)

The applicable range of this calculation method mainly depends on the value of the characteristic parameter L. When L is lower than 120, i.e., the mass transport is essentially limited by surface barriers or both surface barriers and intracrystalline diffusion[12,14], the surface permeability can be well predicted by Supplementary Eq. (3).[12] Correspondingly, the temporal resolution of the uptake rate measurement is about ~1 s, which can be achieved by most of the common-used apparatus, such as

IGA (this work, 1 s)[12], TA instruments (0.5 s)[15], tapered element oscillating microbalance (TEOM, 0.5 s)[16], and infrared (IR, 1 s)[12] spectroscopy. When L is greater than 120, the mass transfer is dominated by intracrystalline diffusion and surface barriers play a negligible role.[12] In this case, it is hard to reliably derive surface permeability of guest molecules via Supplementary Eq. (3) based on the data obtained with the common-used apparatus such as IGA, TA, TEOM and IR spectroscopy since the temporal resolution is not low enough.[12]

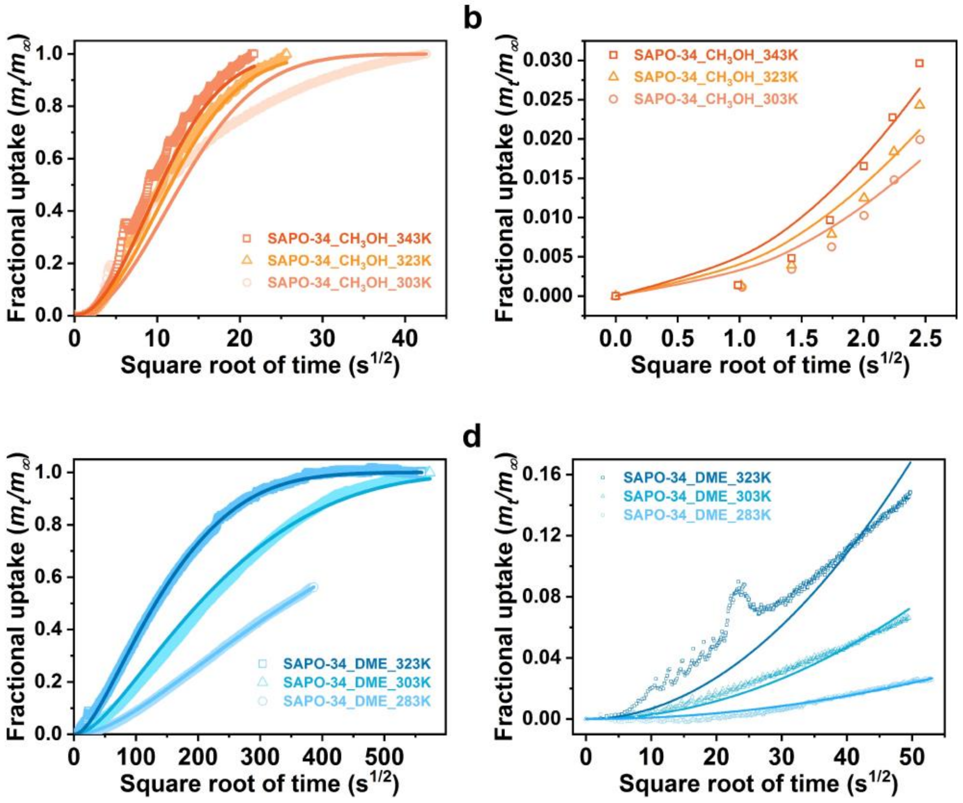

Figure S3. Uptake of methanol and DME in SAPO-34 zeolites at different temperature. (a and c) Uptake rate of methanol (a) and DME (c) in SAPO-34 measured by IGA at 303, 323 and 343 K for methanol and 283, 303 and 323 K for DME under 0.06 mbar. Such low pressure is adopted to ensure that the molecular loading is sufficiently low, where the intracrystalline (transport) diffusivity (D) and intracrystalline self-diffusivity are coincided, considering that the intracrystalline diffusivity of guest molecules is determined by interaction of guest molecule with the host rather than by drag effects with other molecules at sufficiently low molecular loading[9,17,18]. The scatters represent the experimental data while solid lines are fitting results by Supplementary Eq. (5). (b and d) Initial uptake rate of methanol in (a) and DME in (d). The scatters represent the experimental data while dash lines are fitting results by Supplementary Eq. (3). Note that the saturated adsorption mass of DME at equilibrium state under 0.06 mbar at 283 K was obtained by extrapolating the data at 303 and 323 K, due to the super long equilibrium time of DME in this test conditions. The corresponding obtained intracrystalline diffusivity and surface permeability of methanol and DME are shown in Table S1.

Table S1. Intracrystalline diffusivity (D) and surface permeability (α) of methanol and DME on SAPO-34 at 283-343 K obtained from uptake rate of methanol and DME presented in Fig. S3.

Temperature (K)	D ($m^2 s^{-1}$)		α ($m s^{-1}$)	
	CH_3OH	DME	CH_3OH	DME
283	-	9.95×10^{-18}	-	1.86×10^{-11}
303	1.00×10^{-12}	2.85×10^{-17}	5.61×10^{-9}	5.76×10^{-11}
323	2.15×10^{-12}	6.75×10^{-17}	6.89×10^{-9}	1.33×10^{-10}
343	3.00×10^{-12}	-	8.61×10^{-9}	-

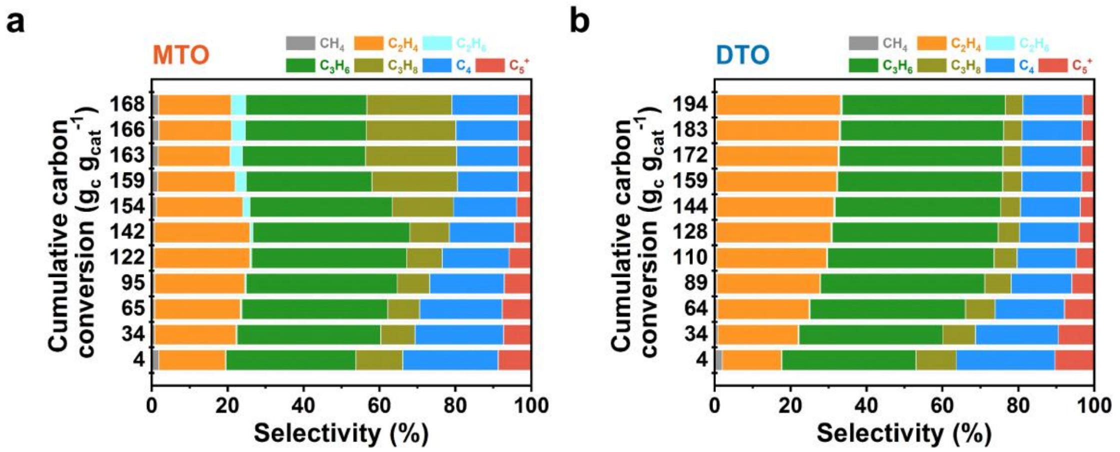

Figure S4. Product selectivity versus cumulative carbon conversion for MTO and DTO reactions over SAPO-34 at 623 K, feeding with equimolar carbon mole (0.0062 mol_{CH2} h^{-1}) of methanol and DME.

Supplementary Note 2. Calculation details of the relative effectiveness factor and relative effective diffusivity of reactant molecules.

The catalyst effectiveness factor (η) is expressed according to Supplementary Eq. (8)[19] below:

$$\eta = \frac{k_{app}}{k}$$

(Supplementary Equation 8)

where k_{app} (s^{-1}) and k (s^{-1}) represent the apparent and intrinsic rate constant, respectively.

Assuming that olefin formation during the MTO and DTO reactions is a pseudo-first order reaction, the apparent rate constant k_{app} was calculated by Supplementary Equation 2 on a carbon basis:[20]

$$k_{app} = -\ln(1 - X)\frac{F_{A0}\rho_C}{C_{A0}W}$$

(Supplementary Equation 9)

where X is the conversion of reactant, F_{A0} (mol s^{-1}) is the molar flow rate of the reactant, ρ_c (g m^{-3}) is the catalyst density, C_{A0} (mol m^{-3}) is the initial reactant concentration calculated from the ideal gas law, and W (g) is the catalyst weight.

The MTO and DTO reactions can be expressed as follows simplified autocatalytic reaction scheme:[21,22]

$R + R \xrightarrow{k_1} P$ (1)

$R + P \xrightarrow{k_2} P$ (2)

where R is reactant; P is hydrocarbon product; k_1 is the intrinsic rate constant of reaction (1) representing direct coupling of methanol or DME to generate initial hydrocarbons; k_2 is the intrinsic rate constant of reaction (2) representing the autocatalytic reaction of methanol/DME with generated hydrocarbons as the active intermediates. After the induction period, the autocatalytic reaction (2) dominates the transformation of the reactant. Therefore, it is reasonable to assume that the intrinsic rate constant k is constant during the MTO and DTO reactions.

Accordingly, combining Supplementary Eq. (8 and 9), one can obtained:

$$\eta_2 = \frac{\ln(1 - X_2)}{\ln(1 - X_1)} \eta_1$$

(Supplementary Equation 10)

Thiele modulus (ϕ), which is a dimensionless number defined by Supplementary Equation 11[23]:

$$\phi = R(\frac{kC_{As}^{n-1}}{D_{eff}})^{1/2}$$

(Supplementary Equation 11)

where *R* (m) is radius or other applicable diffusion length of the catalyst crystal, C_{As} the reactant concentration at the active sites (mol m^{-3}), *n* the order of the reaction (dimensionless), *k* the intrinsic rate constant (unit of *k* depends on *n*), D_{eff} (m^2 s^{-1}) is the effective diffusivity of the reactant in the catalyst.

Assuming that olefin formation during the MTO and DTO reactions is a pseudo-first order reaction, the expression of ϕ can be simplified according to Supplementary Eq. (12):

$$\phi = R(\frac{k}{D_{eff}})^{1/2}$$

(Supplementary Equation 12)

The relationship between η and ϕ is expressed as follows[23]:

$$\eta = \frac{\tanh \phi}{\phi}$$

(Supplementary Equation 13)

Since $tanh\ 2.7 = 0.991 \approx 1$, when Thiele modulus $\phi > 2.7$ or effectiveness factor $\eta < 0.37$, there is:

$$\eta \approx \frac{1}{\phi} \quad (\phi > 2.7\ or\ \eta < 0.37)$$

(Supplementary Equation 14)

As shown in Fig. 3b, when the catalyst effectiveness factor of MTO and DTO reaction was less than 0.37, combining Supplementary Eq. (8, 9, 10, 12 and 14), one can obtained:

$$D_{eff-2} = \frac{\ln(1 - X_2)^2}{\ln(1 - X_1)^2} D_{eff-1}$$

(Supplementary Equation 15)

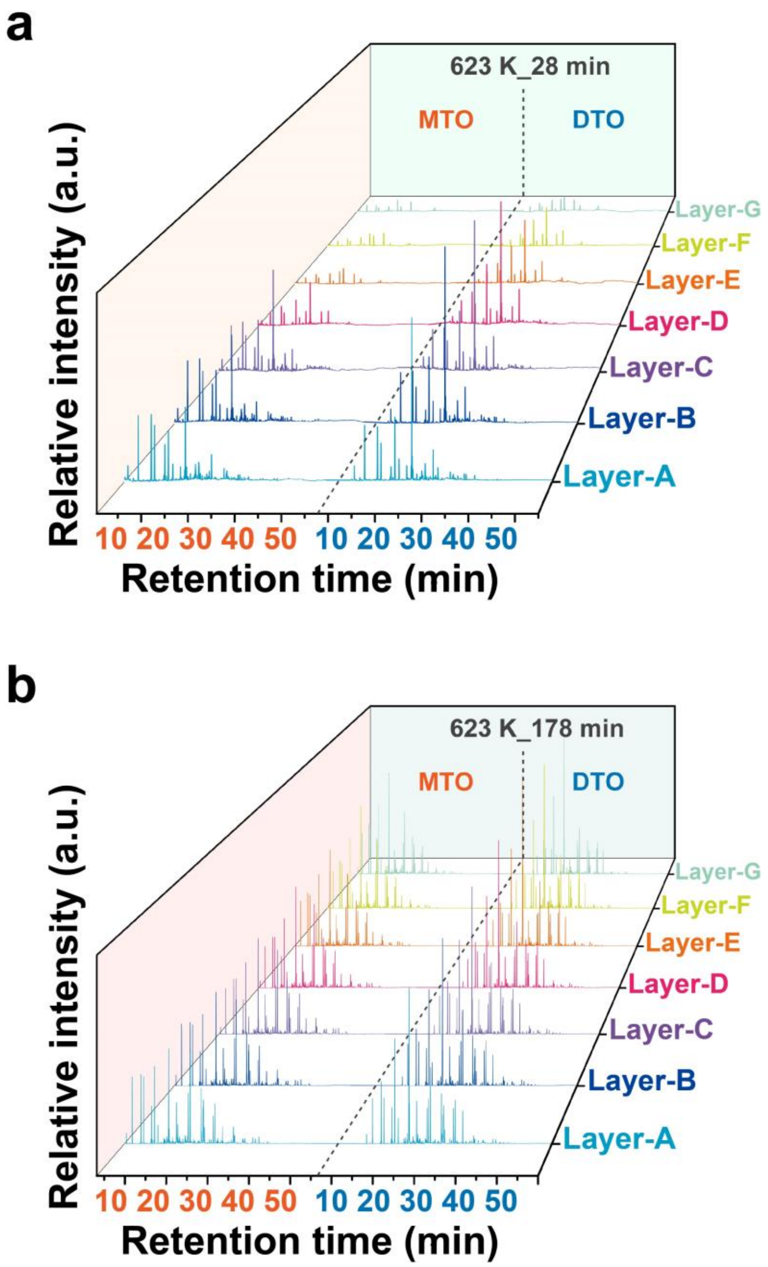

Figure S5. GC-MS total ion chromatograms of organic species confined in seven SAPO-34 catalyst bed layers (Layer-A to Layer-G) after methanol and DME conversions at 623 K, respectively, for 28 min (a) 178 min (b). All peaks are normalized relative to the internal standard peak C_2Cl_6, indicated by * in the chromatograms.

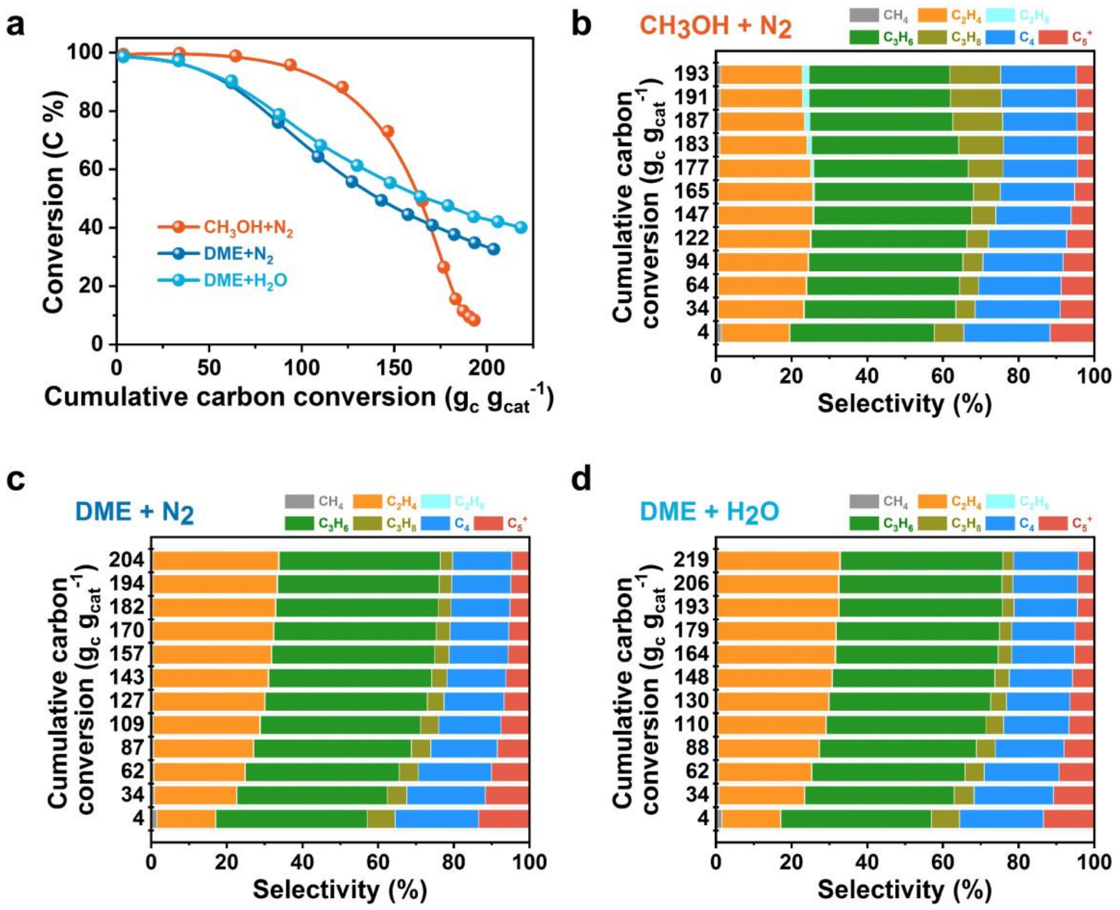

Figure S6. Conversion and product selectivity versus cumulative carbon conversion for CH_3OH-N_2, DME-N_2, and DME-H_2O co-feeding reactions, respectively, over SAPO-34 at 623 K with equimolar carbon of methanol and DME feeding (0.0062 mol_{CH2} h^{-1}). The molar ratio of DME to water is 1:1. H_2O (ultrapure grade) was fed by passing nitrogen through a saturation evaporator kept at 305 K, achieving equal relative partial pressure to DME (4.5%) and half of methanol (9%), and correspondingly, equal flow rate of N_2 was fed to achieve same contact time.

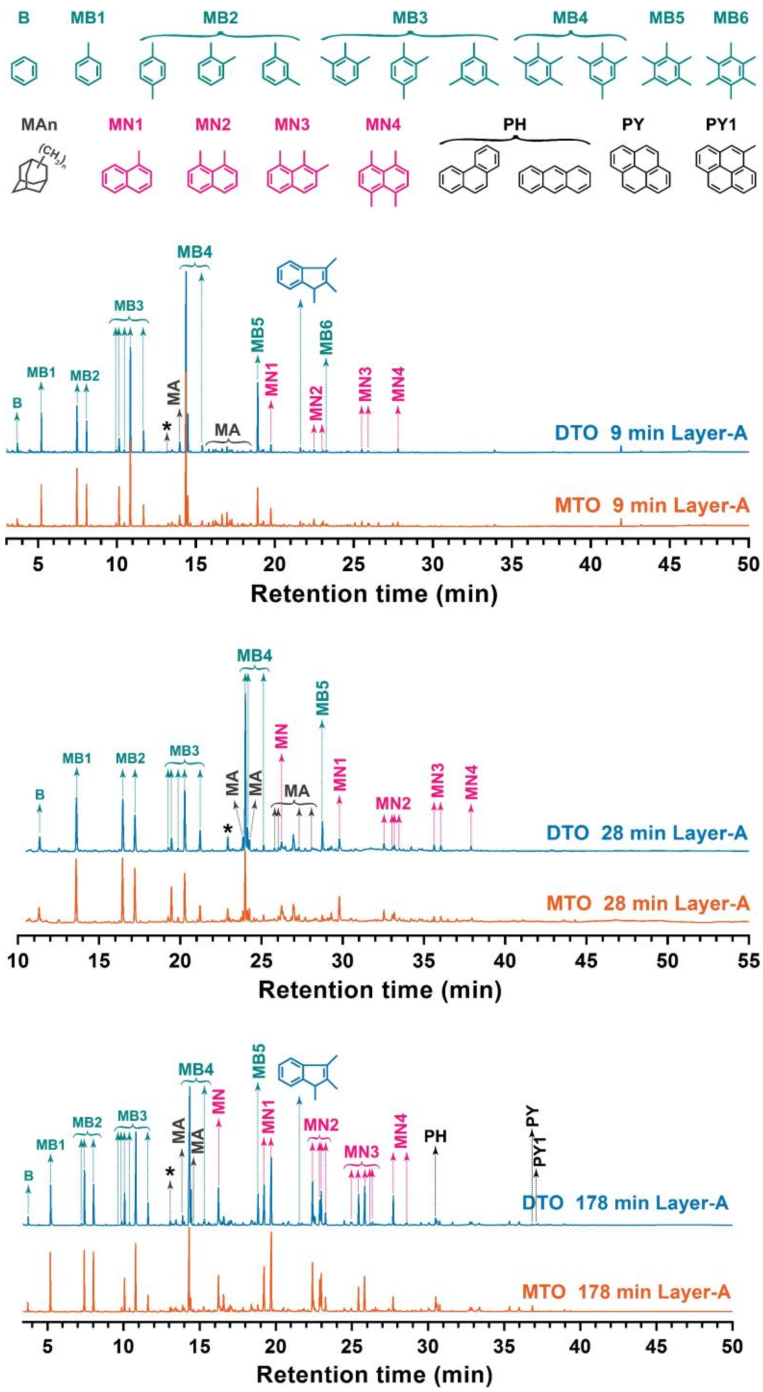

Figure S7. GC-MS spectra of the soluble carbonaceous species retained in SAPO-34 after MTO (representative by Layer-A) and DTO (representative by Layer-A) reaction for 9 min, 28 min, and 178 min over SAPO-34 at 623 K. The confined species for all samples mainly include abundant methylbenzene (MB) and methylnaphthalene (MN), and slight methyladamantane (MA), phenanthrene (PH) and pyrene (PY). All peaks are normalized relative to the internal standard peak C_2Cl_6, indicated by * in the chromatograms. Reaction conditions are the same as that in Fig. 3a.

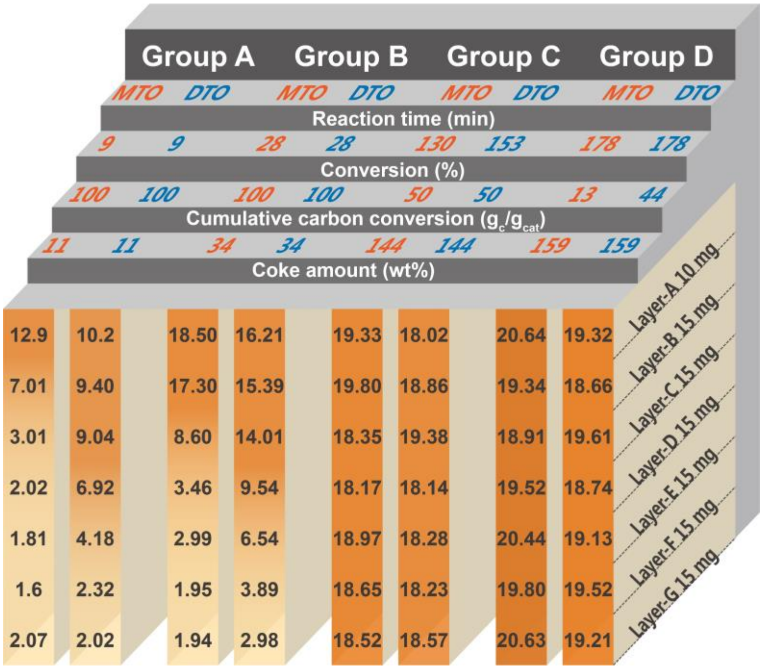

Figure S8. The amount of coke formed in seven SAPO-34 catalyst bed layers after methanol and DME conversions at 623 K, respectively, for various time on stream.

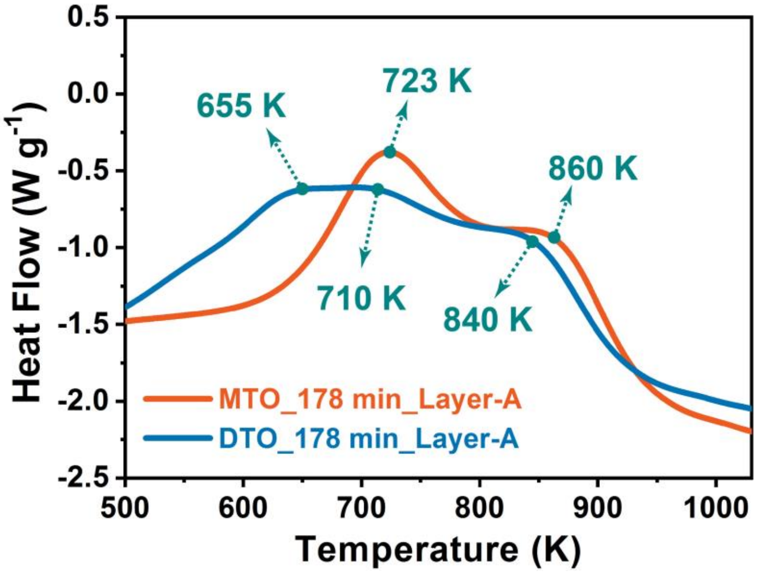

Figure S9. Temperature programmed oxidation (TPO) profiles of spent catalyst from the Layer-A SAPO-34 catalysts by after MTO and DTO reactions at 623 K for 178 min. Reaction conditions are the same as that in Fig. 3a.

As shown in Fig. S9, the higher exothermic temperature for MTO than DTO indicated that the coke in MTO is heavier than that in DTO after 178 min reaction.

REFERENCE

1. Chen J, Li J and Wei Y *et al.* Spatial confinement effects of cage-type SAPO molecular sieves on product distribution and coke formation in methanol-to-olefin reaction. *Catal Commun* 2014; **46**: 36-40.
2. Gao S, Xu S and Wei Y *et al.* Direct probing of heterogeneity for adsorption and diffusion within a SAPO-34 crystal. *Chem Commun* 2019; **55**: 10693-6.
3. Wong YC, Ysselstein D and Krainc D. Mitochondria-lysosome contacts regulate mitochondrial fission via RAB7 GTP hydrolysis. *Nature* 2018; **554**: 382-6.
4. Qi Q, Chi W and Li Y *et al.* A H-bond strategy to develop acid-resistant photoswitchable rhodamine spirolactams for super-resolution single-molecule localization microscopy. *Chem Sci* 2019; **10**: 4914-22.
5. Frisch M J T, G. W.; Schlegel, H. B.; Scuseria, G. E.; Robb, M. A.; Cheeseman, J. R.; Scalmani, G.; Barone, V.; Mennucci, B.; Petersson, G. A.; Nakatsuji,H.; Caricato,M.; Li, X.; Hratchian, H. P.; Izmaylov, A. F.; Bloino, J.; Zheng, G.; Sonnenberg, J. L.; Hada, M.; Ehara, M.; Toyota, K.; Fukuda, R.; Hasegawa, J.; Ishida, M.; Nakajima, T.; Honda, Y.; Kitao, O.; Naka, H.; Vreven, T.; Montgomery, J. A.; Peralta, J. E.; Ogliaro, F.; Bearpark, M.; Heyd, J. J.; Brothers, E.; Kudin, K. N.; Staroverov, V. N.; Kobayashi, R.; Normand, J.; Raghavachari, K.; Rendell, A.; J. Burant, C.; Iyengar, S. S.; Tomasi, J.; Cossi,;M.; Rega, N.; Millam, J. M.; Klene, M.; Knox, J. E.; Cross, J. B.; Bakken, V.; Adamo, C.; Jaramillo, J.; Gomperts, R.; Stratmann, R. E.; Yazyev, O.; Austin, A. J.; Cammi, R.; Pomelli, C.; Ochterski, J. W.; Martin, R. L.; Morokuma, K.; Zakrzewski, V. G.; Voth, G. A.; Salvador, P.; Dannenberg, J.J.; Dapprich, S.; Daniels, A.D.; Farkas, O.; Foresman, J. B.; V.Ortiz, J.; Cioslowski, J.; Fox, D. J. Gaussian 09, Revision B.01, Gaussian, Inc.: Wallingford, CT, 2010.
6. O'Malley PJ and Dwyer J. Ab-initio molecular orbital calculations on the siting of aluminium in the Theta-1 framework: some general guidelines governing the site preferences of aluminium in zeolites. *Zeolites* 1988; **8**: 317-21.
7. Chai J-D and Head-Gordon M. Long-range corrected hybrid density functionals with damped atom–atom dispersion corrections. *Phys Chem Chem Phys* 2008; **10**: 6615-20.
8. Sastre G, Catlow CRA and Corma A. Diffusion of benzene and propylene in MCM-22 zeolite. A molecular dynamics study. *J Phys Chem B* 1999; **103**: 5187-96.
9. Kärger J, Ruthven DM and Theodorou DN. *Diffusion in Nanoporous Materials.* Weinheim: Wiley-VCH, 2012.
10. Crank J. *The Mathematics of Diffusion.* London: Oxford University Press, 1979.
11. Fasano M, Humplik T and Bevilacqua A *et al.* Interplay between hydrophilicity and surface barriers on water transport in zeolite membranes. *Nat Commun* 2016; **7**: 12762.
12. Gao M, Li H and Yang M *et al.* Direct quantification of surface barriers for mass transfer in nanoporous crystalline materials. *Commun Chem* 2019; **2**: 43.
13. Remi JCS, Lauerer A and Chmelik C *et al.* The role of crystal diversity in

understanding mass transfer in nanoporous materials. *Nat Mater* 2016; **15**: 401-6.
14. Heinke L. Significance of concentration-dependent intracrystalline diffusion and surface permeation for overall mass transfer. *Diffus Fundam* 2007; **4**: 12.1-11
15. Humplik T, Raj R and Maroo SC *et al.* Effect of hydrophilic defects on water transport in MFI zeolites. *Langmuir* 2014; **30**: 6446-53.
16. Chen D, Rebo HP and Holmen A. Diffusion and deactivation during methanol conversion over SAPO-34: a percolation approach. *Chem Eng Sci* 1999; **54**: 3465-73.
17. Chmelik C, Bux H and Caro J *et al.* Mass transfer in a nanoscale material enhanced by an opposing flux. *Phys Rev Lett* 2010; **104**: 085902.
18. Beerdsen E, Dubbeldam D and Smit B. Understanding diffusion in nanoporous materials. *Phys Rev Lett* 2006; **96**: 044501.
19. Haag WO, Lago RM and Weisz PB. Transport and reactivity of hydrocarbon molecules in a shape-selective zeolite. *Faraday Discuss Chem Soc* 1981; **72**: 317-30.
20. Chen D, Rebo HP and Moljord K *et al.* Dimethyl ether conversion to light olefins over SAPO-34: deactivation due to coke deposition. *Stud Surf Sci Catal* 1998; **119**: 521-26.
21. Ono Y and Mori T. Mechanism of methanol conversion into hydrocarbons over ZSM-5 zeolite. *J. Chem Soc Faraday Trans 1* 1981; **77**: 2209-21.
22. Lin S, Zhi Y and Chen W *et al.* Molecular routes of dynamic autocatalysis for methanol-to-hydrocarbons reaction. *J Am Chem Soc* 2021; **143**: 12038-52.
23. Peng P, Gao X-H and Yan Z-F *et al.* Diffusion and catalyst efficiency in hierarchical zeolite catalysts. *Natl Sci Rev* 2020; **7**: 1726-42.
